# Students’ Experiences in Suddenly Transformed Living and Educational Environments by COVID-19

**DOI:** 10.3389/fpsyg.2021.782433

**Published:** 2021-11-08

**Authors:** Fernando Hernández-Hernández, Juana M. Sancho-Gil

**Affiliations:** ^1^Department of Visual Arts and Design, University of Barcelona, Barcelona, Spain; ^2^Department of Didactics and Educational Organization, University of Barcelona, Barcelona, Spain

**Keywords:** learning lives, online learning, emotions, adaptation, agency, COVID 19

## Abstract

This manuscript builds on research about how university students felt affected by the Covid19 pandemic and, especially, by the irruption of non-face-to-face classes and mixed teaching methods in this context. How have young people experienced this situation? How has it affected their wellbeing and the learning strategies should develop have had to incorporate into their virtual relationships? their virtual relationships? How have they related and relate to virtual tools for a task that they have always experienced face-to-face? To answer these questions, the TRAY-AP project that investigates how university students learn collected 89 scenes that show the effects of the Covid 19 on their lives and the university. We grouped these scenes into seven key concepts to detect how students were emotionally affected, especially by moving from face-to-face to virtual learning. From this analysis, although primarily negative, the emotional effects have also allowed them to generate positive strategies for readaptation and collaboration with other colleagues. All of which opens the way to rethink the predominant pedagogical and knowledge relations in the university.

## Introduction

The abrupt and profound changes brought about by the Covid 19 pandemic have profoundly transformed people, families, institutions, and societies’ ways of life worldwide. Situations provoked by this context are having considerable consequences and effects on people mental health (Giuntella et al., 2021), especially in the emotional and affective dimensions due to the imposed “social distance” (Sikali, 2020) and the need to reshape personal and professional settings (Strom and Gumbel, 2021). In this situation, higher education students attending face to face institutions had to unexpectedly adapt to new learning environments in which knowledge, affects, contact, bodies, and complicities were placed in an unknown dimension (Farnell et al., 2021; Pokhrel and Chhetri, 2021). Both for them, teachers and administrators.

This manuscript gives an account of research about how higher education students are experiencing the changes in their lives caused by the pandemic and, especially, by the irruption of online teaching and the use of mixed teaching methods in the context of Covid19. The University of Barcelona, like many others, made a quick adaptation to the new situation. Each teacher used the resources at hand to ensure that no student was left unattended. However, how have young people experienced this situation? Has this new scenario affected their wellbeing and the learning strategies they have had to incorporate into the relationships situated in the virtual terrain? How, in short, have they related and related to virtual tools to carry out tasks they have always experienced in a face-to-face setting. These and other issues have risen to the top of the agendas of many researchers in different countries. Understanding how students shape their experiences in this ambiance is relevant for assessing the situation we find ourselves in and making decisions that bring these exceptional circumstances closer to students’ needs.

### State of the Art

In April 2020, we received an invitation from the Universidad Autónoma de Chile to participate in/advise a research project entitled “Didactic-organizational, bodily and emotional factors that, in a non-classroom context and in times of pandemic, contribute to meaningful learning of university students”. Collaboration with the preparation of this project made us realize that studies were underway – early in the pandemic – showing that students worldwide have been affected by the spread of Covid-19. The main reasons were travel restrictions, the physical distancing, the isolation in their residences and the closing of borders. These new circumstances affected their life plans and priorities and their interest in online classes (Quacquarelli Symonds, 2020a,b).

Since the pandemic’s beginning, there have been many publications in different countries (Crawford et al., 2020). Among them, we find those analyzing the problems and challenges of this new situation (Quintana, 2020; Tejedor et al., 2020; Tesar, 2020; Toquero, 2020) such a sensitive issue as student assessment (García-Peñalvo et al., 2020); showing the emerging vulnerabilities in education systems (Ali, 2020); analyzing the opportunities and threats stemming from remote learning (Ślaski et al., 2020); offering world’s perspective on the impact of COVID 19 in higher education (Aristovnik et al., 2020; Bairagi, 2020; Marinoni et al., 2020).

Studies about the impact on students’ lives focused on their experience in the distance learning model (Adnan and Anwar, 2020; Baladrón Pazos et al., 2020; Pérez-López et al., 2021); on various aspects of their lives on a global level (Aristovnik et al., 2020); or different aspects of their emotional health, stress and wellbeing (Apaza et al., 2020; Bono et al., 2020; Nurunnabi et al., 2020; Garvey et al., 2021; Holzer et al., 2021; Rodríguez-Larrad et al., 2021; Van de Velde et al., 2021).

These early studies and those carried out the following year (Farnell et al., 2021; Pokhrel and Chhetri, 2021) showed that quarantining at home and the closure of university facilities were the main reasons why students felt disconnected from society and their social circles (Killian, 2020). In some cases, students reported negative experiences of having to return to the family home during the pandemic due to family environments that were not conducive to online learning (Killian, 2020). The detection of these initial reactions opened several studies on students’ psychological stress and distress. One example was the study by Arënliu and Bërxulli (2020), who measured the psychological pain of students at the University of Pristina in the early days of the Covidien-19 pandemic in Kosovo. These authors found significant differences among students in their motivation to attend online lessons and their levels of psychological distress. Students reported that they were not motivated to participate in online teaching and showed high (moderate to severe) levels of psychological distress, in contrast to students who reported being highly motivated to attend online lessons. It should be kept in mind that these results may have changed in later stages of physical distancing (Arënliu and Bërxulli, 2020) and that there may be an increase in stress levels with prolonged social isolation or quarantine (Brooks et al., 2020 to Arënliu and Bërxulli, 2020). Within this line of studies on students’ experiences, one of the most relevant to the breadth of the sample is the one that has focused on how the pandemic has affected students’ wellbeing and lives (Van de Velde et al., 2020). This study involved students from 27 European countries, those from North America and South Africa.

Following these studies, the results of the UNESCO (2020) report suggest that, globally, the main concerns of university students are social isolation, financial problems, internet connectivity and, in general terms, pandemic-related anxiety. In Latin America, however, the hierarchy of concerns is different. UNESCO’s Chairs have prioritized three areas: Internet connectivity, financial issues, and difficulties in maintaining a regular timetable associated with forms of teaching and learning in schools that do not encourage self-regulated learning.

In summary, studies on how pandemic-generated situations in the lives of university students have shown that institutions that respond to both emotional and academic aspects favor better academic performance and learning engagement. In this sense, universities that support students’ wellbeing also increase students’ feelings of belonging within the education system. With this dual support, students feel safer and more satisfied with each other and acquire more appropriate coping and stress management skills (Sadock et al., 2009; Kieling et al., 2011; Hyseni Duraku and Hoxha, 2018, 2020). These authors’ contributions indicate that further emotional support for students has become apparent during this exceptional university period. In this regard, students have stated that universities can play an essential role in social isolation by communicating with them and providing emotional support during these difficult times (Quacquarelli Symonds, 2020b).

Concerning another issue we address in our research: how online learning has affected students’ wellbeing, different studies (Cidral et al., 2018; Selvaraj, 2019) have evidenced that user satisfaction and e-learning systems significantly impact students’ success. Against this backdrop, in the context of Covid, the study by Quacquarelli Symonds (2020b) indicates that while some students reported that they were enjoying online teaching, others, due to university closures, expressed a lack of motivation and negative attitudes toward online learning. However, as we intend to do in our study, it is necessary to see whether this dissatisfaction occurs if it is a modality imposed by exceptional circumstances and not a learner’s choice. Within this theme, the research by Shahzad et al. (2021) carried out to analyze the impact of e-learning on the learning of women and men at university shows that women make more use of the applications and possibilities related to e-learning platforms.

Regarding e-learning, UNESCO (2020) shows that the abrupt entry into a complex teaching modality, with multiple technological and pedagogical options, and a steep learning curve, can generate less optimal results, frustration, and anxiety. The main challenge being the adaptation to an educational modality never experienced before without the corresponding training. Coronateaching refers to an emerging phenomenon with psycho-affective implications for both teachers and students to describe this teaching modality. These implications would be like a syndrome experienced by the teacher or student when feeling overwhelmed by receiving excessive information through educational platforms, mobile applications, and email. To this can be added frustration and helplessness stemming from limitations in connectivity or lack of technical knowledge for the operation of digital platforms and resources (UNESCO, 2020, p. 25).

Almost all studies consisted of an online survey (Khan, 2021), hence the need to look at more embodied experiences. Research in which students can openly explore their feelings, moments, anxieties, discoveries, and hopes, all of whom report on their subjective wellbeing.

### Situating TRAY-AP Research Project

Building from the TRAY-AP^1^ project, this paper explores students’ experiences from a more personalized and closer perspective. The TRAY-AP project aims to reveal the learning trajectories of university students, considering their conceptions, strategies, technologies, and learning contexts. To inquire about students’ learning lives, we adopt two notions as conceptual guides. The first one is “a person-in-context perspective” (Turner and Patrick, 2004, p. 1764) linked to the referent “persons-acting-in-setting” as the unit of analysis (Lave, 1988, p. 189). The second follows the differentiation that Biesta (2013) makes between “socialization,” which concerns how, through education, individuals become part of existing orders and traditions, and “subjectification” that has to do with ways of being not determined by those orders and traditions.

The TRAY-AP research project departs from an onto-epistemological and methodological position based on a relational and performative ethic (Geerts and Carstens, 2019). This position implies considering the “Other” as a “being in becoming” who is a bearer of knowledge and experiences. In the research, participants can show themselves as becoming subjects in their relationships with learning and knowledge.

In this article, the guiding question is: how do students participating in research -taking place during the COVID pandemic- live, dialogue, and incorporate into their learning lives an experience that disrupts their lives and the “contextures” that shape their learning in college and beyond?

We have proposed a research project within this conceptual and methodological framework in which young people make their “learning lives” visible.

Fifty participants from Catalonia (28) and the Basque Country (22) are taking part. Of these, 30 are women, and 20 are men, a proportion close to the distribution observed in Spanish universities in the 2019–2020 academic year (55.6 and 44.4). By branches, we selected them following the distribution that appears in Catalonia and the Basque Country in the report of the Ministry of Universities (Ministerio de Universidades, 2021): Social and Legal Sciences 18, Arts and Humanities 12, Engineering and Architecture 5, Experimental Sciences 6, Health Sciences 6, and double degrees 3 (Table 1). This sample responds to the “qualitative” nature of the research and the demanding intensity of monitoring and analyzing each learning life. It is also like that found in other research with a similar focus. For example, 48 students participated in Jornet and Erstad’s (2018) study and 44 in the study by Biasin and Evans (2019).

**TABLE 1:** TRAY-AP research sample.

**Universities**	**Number of students**	**Knowledge areas**
Barcelona, Girona, Catalan Polytechnical University, Autonomous University of Barcelona, Pompeu Fabra (28) Basque Country (22)	30 women 20 men	Social and legal sciences (18), arts and humanities (12), engineering and architecture (5), experimental sciences (6), health sciences (6), double degrees (3)

Considering this research’s contextual and corporeal nature, invited participants should have a high predisposition to collaborate. They should agree to spend several hours with researchers and prepare documents to share. Researchers, on their side, should be able to contact personally participants on at least four occasions. These kinds of processes are only possible within a certain proximity. This was the reason for working with students from the two Autonous Comminities (Catalonia and the Basque Country) to which the research teams belong. That does not question, however, the research rigorousness and validity. From an onto-epistemological position, which does not aim to make statistical generalizations and test hypotheses based on logical positivism, but to explore a complex, multi-layered phenomenon in which all actors and actants – human, non-human and matter – have a role to play (Latour, 2008; Tuin and Dolphijn, 2012).

The choice of university degrees and the distribution of participant students aim to provide as diversified a sample as possible. The purposive selection of young people follows the chain referral method (Penrod et al., 2003), often used when there are difficulties finding participants due to the sensitive nature of the “object” of study.

## Materials and Methods

We conducted four conversations with each student to meet the project’s objectives and ensure participants’ engagement and interest.

• The first is to explore how different studies portrait contemporary young university students and address questions related to their relationships with learning (Veen and Vrakking, 2006; Seemiller and Grace, 2016; Twenge, 2017; Haidt and Lukianoff, 2018; Carr, 2020; Desmurget, 2020). In this meeting, we asked two main questions, which are the trigger for this article: How are their learning experiences during the pandemic? What are the possibilities and limitations of e-learning?
• In the second meeting, students reconstruct their learning lives based on a biogram (Abel, 1947; Domingo et al., 2017).
• Students share a field diary of learning ‘scenes’ in the third session (Denzin, 1997).
• In the fourth, students comment on questions posed by the researchers in dialogue with the transcripts of previous encounters and on the script of each learning life story.
• A final meeting revolves around the validation of this learning life story. In all encounters, comments and feelings on their experiences during the pandemic also appear.

We transcribed all the conversations and fragmented them into “significant scenes” that made it possible to identify and establish if standard chains of thought, concepts, paths, threads, and conjunctions exist and whether these can suggest an explicit logic of association or a common argument (Beach et al., 2014, paraphrased). We used significant scenes (Denzin, 1997) as an analysis strategy because they allow us to configure the research evidence. The scenes show those singular experiences in that they represent a marking moment that we explored to promote critical reflection (Ornelas, 2016). The notion of scene originates from Denzin (1997), who considers it a fragment of a narrative text that goes beyond the traditional boundaries of the ethnographic text. The basic unit of analysis is not the fact but the scene, the situation in which an event occurs. Stories and poems are written in the facts, not about the facts. […] Personal narrative can also be ethnography. […] These materials (self-dialogue, scenario, and conversation) evidence that the described events are authentic and not fragments of their imagination (Denzin, 1997, 208, paraphrased).

According to Bowie et al. (2003), the adjective “significant” is a general term commonly applied to events or incidents described as critical, adverse, near misses, or errors. As Charmaz (2006) points out, meaningful scenes allow the freezing and lengthening of time so that it is possible to dialogue with them. They “confront us with new situations or show us other ways of doing and other types of relationships” (Hernández-Hernández, 2007, p. 182). They allow us to embody the localization and partiality from which this text emerges.

We located the scenes in conversations with 14 collaborators (10 women and four men) who made comments related to (a) their emotional and wellbeing/discomfort during the pandemic; and (b) the relationship with e-learning and the perceived emotional effects on the quality of their learning.

### Analysis and Definition of Key Concepts

Taking as a reference a previous study (Hernández-Hernández and Sancho-Gil, 2017) on the learning experiences of young people in secondary education, we organized the dialogue with the scenes in the following steps: (1) Reading carefully and collaboratively in pairs the 14 collaborators’ transcriptions. (2) Selecting all the fragments of each report related to scenes referred to their emotional experiences in life and virtual learning. In this process, we located 89 scenes. (3) Analyzing each scene by pairs to check key emotional concepts and validate their relevance as a foundation for making general claims and qualitative similarities and differences. (4) Challenging and supporting the relevance of the attribution of the seven key concepts identified in the scenes, assuming the liminality of some attributions. (5) Dialogue with the scenes, grouped into crucial concepts and relate them to bibliographic references.

As shown in Table 2, we named seven key concepts after carefully and rigorously analyzing each scene and discussing their most significant features. The identified key concepts are:

**TABLE 2:** Extract of learning scenes organization.

**Student name and affiliation**	**Scene**	**Focus**	**Emotional affect**
Anna (Medicine, UB)	28. The second semester of 2020–2021 is beginning, and we are about to close the first anniversary of the Covid pandemic. Most of the classes are still distance learning. This situation leads to intensive use of the digital resources available, including the drive organized by themselves and the UB’s Virtual Campus.	In the second academic year of COVID, students have learned to organize learning collectively and on their initiative, in parallel to the teachers’ proposals.	Positive effects: (3^1^) Facilitate students’ self-organization
Mikel (Computer engineering, UPV)	34. The virtual teaching they have been giving us during the confinement and Covid are negatively allied. With lockdown, virtual teaching was a very limiting factor. Face-to-face classes are not the same as having a PowerPoint and talking without asking questions until the end. There is a lack of contact, the learning situation changes. It is not the same to be in a classroom in a work environment, in a study environment, as it is at home where you have I don’t know how many distractions.	The virtuality of the classes generates isolation and reduces the interaction with the group and the teachers, “and they are negatively allied”.	Virtual classes generate adverse effects (4) and are a limiting factor in students’ lives.
Anastasia (UPV, Philosophy)	64. Well, we have had many classes and so on, but. on the one hand, is your ability to concentrate, if you care, you go to the class and listen to it. You learn the same as if you were here because, in the end, they are explaining it to you anyway, what you learn more or less, in the end, depends on your ability to concentrate and how much you want to put in on your part.	If you care, you go to the virtual class, listen to it, and learn the same.	Ambivalent effects: (3) The concentration depends if the student cares, not about virtual conditions.

*^1^It indicates the number of times it is pointed out.*

# Wellbeing versus discomfort (23^2^)

It refers to scenes in which they point out in a general way how the pandemic generated situations of wellbeing or discomfort in life in general, as well as in the university.

I think I’m living it pretty badly, and now I’m not tragic, but the first lockdown was indeed much worse (Marc, student of Physics).

# Interactions with other people (10, 1 mix)

They refer to interactions and exchanges with teachers, students, and family members and how they were affected by the pandemic situation.

It reduces the interaction between the one who explains or teaches and the one who receives the knowledge so much that it is practically impossible. I won’t say it’s unfeasible, but it becomes very complicated (Marc, student of Physics)

# The role of corporeality (7, 1 mix)

Grosz (1994, 2004) called corporeality the notion that displaces the body toward the non-human, more-than-human, beyond-human and concentrates on the zones of proximity between the body and the world.

It’s true that at least one thing that many professors tell us, and I understand it perfectly, is that I don’t see your face and I don’t see if you don’t understand anything if you understand everything and I have to go faster (Marc, student of Physics).

# The emotional effect of virtual teaching and learning (35)

The pandemic changed the terrain of the face-to-face and virtual pedagogical relationship. This alteration entailed a reconfiguration of the presence of bodies and led to configure the computer screen as a “place” of learning, presences and absences. This unexpected change affected students and teachers. This concept refers to the ways of feeling affected and was reflected in the most significant number in the students’ scenes. That is why we pay special attention to it in the following section.

What annoyed me the most was having to break the link suddenly (at her internship). It is true that later, with the virtual networks and so on, we tried to keep in touch again, with the mothers, the educators and so on, and it was a way of learning virtually (Nerea, student of Social Education).

Yes, it has made me lose the desire to learn or to do something I like, and I have simply been a bit of a robot. They told me something, they explained something to me, and I was doing it step by step (Lisa, student of Architecture).

# Emotional Learning time (6)

The pandemic changed the terrain of the face-to-face and virtual pedagogical relationship. This alteration entailed a reconfiguration of the presence of bodies and led to configure the computer screen as a “place” of learning, presences and absences. This unexpected change affected students and teachers. This concept refers to the ways of feeling affected and was the one Confinement and virtual lessons altered time management and the relationship with time. This concept relates to how the effects of time change in the emotional life of students.

The timetable says that from 8 to 10 am I have to do 2 h of dermatology; from 10–12 infectious diseases. and then you organize yourself as you can or want to. For example, I used to have lessons from 2 to 7 pm, and in the morning you could do whatever you wanted. Now we do all the theory classes in 1 month, in February. And then they cram in 8 h of classes a day so that you can do practical work (Anna, students of Medicine).

# Personal living strategies (4)

Lockdown meant a disruption in outdoor life, something fundamental in Mediterranean countries. Students had to develop strategies in everyday life to adapt to this new situation. This concept refers to strategies aimed, in most cases, at achieving better emotional accommodation.

So, I don’t know. I learned many strategies. I’ve been going to a psychologist for a long time, and it’s helped me understand that a space doesn’t determine how I am and how I am. I also adopted a dog, and that does a lot. Right now, if I found myself in confinement like the other one, I think I would be pretty calm, and I would have resources, and I would go on with my normal, quiet life. Before, I was caught off guard amidst construction work. It was “madness” (Maria, Fine Arts, student).

# Emotional space (5)

Because of the disruption of life outside, household spaces had to be reorganized. Houses were not designed for many household members to live and work in, and the adaptation had emotional effects.

On top of that, many things came together. We had half-finished work in the kitchen, and we had to [prepare] food with a microwave oven. I didn’t imagine that this would “crush” me so much mentally. My mother, a shop cashier, came home every day “pulling her hair out” without a kitchen (Maria, Fine Arts, student).

### The Emotional Effects of Virtual Teaching and Learning in Pandemic Times

Participants in our study highlighted the “emotional effect of virtual teaching and learning” as the most salient in their learning lives during the period they suffered from movement restrictions and university closure. We, therefore, focus the second level of analysis and results on this key concept.

Khan (2021, p. 8) argues, based on his review of 39 studies that focused on the impact of the COVID-19 pandemic, that the abrupt closure of higher educational institutions, followed by a lockout, has left individuals bewildered and dealing with a variety of difficulties. This situation can contribute to increased anxiety and stress, such as job instability, financial worries, homeschooling, despair, loneliness, loss, trauma, and illness. Khan, to confirm this statement, refers to a study by Watermeyer et al. (2021) focused on the online migration that followed COVID-19, the severe damage caused to pedagogical roles in higher education, in terms of dysfunction and disturbance, not only from an educational perspective but also in terms of academics’ personal lives. We want to put this idea of “dysfunction and disruption” in dialogue with the 34 scenes collected in the TRAY-AP study, which in some respects confirms this assumption. However, it also shows how students generate individual and collective responses to develop their agency and resilience. That is to say, their capacity of acting and recovering from difficulties. For Prout and James (1997) (in Rose, 2011, p. 66) “agency is the ability to initiate an action of choice, reflected as creative production where people’s activity can be a source of change.”

To carry out this dialogue, we created a table with four columns. In the first one, we placed the student’s name, the studies s/he is doing, and the university s/he belongs to. We placed the 34 scenes linked to the key concept “emotional effects of teaching and virtual learning” in the second. In the third column, we highlighted the effects that stood out in the scenes. In the fourth, we indicated the positive, negative or ambivalent character, in emotional terms, of those effects. Table 2 shows an example of the organization of the scenes.

A negative emotional effect slows down the student’s agency, leaves them in suspense, prevents them from acting, and produces inhibition, discomfort, and decisiveness. A positive emotional effect leads students to take on challenges, propose collaborative initiatives, and generate strategies that activate their capacity for agency. An ambivalent emotional effect that includes characteristics of the two previous ones moves them in several directions. All this means that the capacity for agency can be slowed down but also activated. To appreciate in detail the characteristics of these effects, we have organized them, inspired by Huguley et al. (2021), in Table 3, which shows each of the effects reported by the students.

**TABLE 3:** Students’ emotional effects on learning during the COVID-19.

**Emotional effect**	**Characterization**	**Relevance**
Negative emotional effect	The time limitation when performing online activities’ make you very nervous’.	Feel nervous
	Replacing teacher interaction with video generates ‘overload on oneself’.	Feel overload
	The substitution of presence for virtuality in parallel groups leads students who are not in front of the teacher to leave the classroom.	Dropout for lack of attention
	The virtuality of the classes generates isolation and reduces the interaction with the group and the teachers, “and they are negatively allied.”	Generates isolation and reduces the interaction with others
	My performance dropped a lot because I was at home, and there were more distractions.	Distraction, drop in performance.
	The personal distance from teachers and classmates, imposed by virtuality, affects the quality of pedagogical relationships.	Affects the quality of relational experiences (3).
	I have made a very strong disconnection from the university, and really in this last year, the fact of being in from the screen, my ability to concentrate has decreased a lot.	Disconnection, lack of concentration, and dispersion.
	I made a change and relaxed and stopped trying so hard to learn. -I’ve learned some things – but it’s been a year of “well: I pass.”	Lack of effort to learn, lack of retention when preparing for an exam.
	I have lost this ability to. well, I don’t think I have lost it, but the fact of being alone, isolated, working from home makes it difficult getting together with my friends to work on a topic.	Loss of ability to learn with others due to isolation.
	It has made me lose the desire to learn or to do something I like, and I have simply been a bit of a robot.	Being “a bit robotic” reduces the intensity of learning and results in loss of agency.
	It is much more violent because of the virtual campus.	Virtuality generates symbolic violence personally and in the group.
	There are people, for example, a classmate, that the pandemic affects her a lot. She is very embarrassed to put the camera on or talk in class; she is very anxious, so we talked more about this topic.	Shame and anxiety in people who are emotionally affected by the pandemic situation.
	Because there are no presential classes, some teachers don’t let me do the projects I want to do as performances or projects with a robust feminist component.	Affects students’ agency and autonomy.
	If everything has to be online, the student has to put in a lot of effort, and it is much easier to get out of the computer.	Online classes demand extra effort for learning and facilitate dropout.
.	The fact that you can comment with someone without having to send a WhatsApp or put in the microphone relaxes you a lot, so the fact of being. isn’t it?	Virtuality goes in a different direction of face-to-face proximity that generates a climate of relaxed proximity.
Positive emotional effects	Recording lectures, as a flipped classroom strategy, build student confidence.	Feel confident.
	The use of the flipped classroom generates the perception of teacher’s satisfaction by the student.	Feel empathy with the teacher.
	Entering the 2nd year of COVID, students have learned to organize learning collectively and on their initiative, in parallel to the teachers’ proposals.	Facilitate student’s self-organization
	I am trying to delve a little deeper into what exactly is going on with the teachers. I conceive it as several lines of action that can complement each other a little bit. everything has to do, doesn’t it?	Promoting alternatives to the limitations generated by teachers in the face of virtuality.
	I have used that video of al-Sa’dawi, and I have used other things, and there began a little bit like the line of work of the university, pandemic and mental health.	Recovering the initiative, establishing relationships, and confronting mental health.
	I was frustrated with the sculpture class. Well, it occurred to me, I started to do this (specific action), the schedule (activities) came to me there. And the schedule, well, I showed it to I don’t know who, who liked it.	Generate alternatives and answers in collaboration with other students in the face of limitations on virtual classes.
	And so, it has been good for me to get to know myself in that situation, set patterns, organize myself, and set routines.	Contribute to self-knowledge
	I pass the memes to my classmates, and they like them, and I don’t know like there is a line of.; we want to continue talking about this topic, which seems positive to me because it is like a more profound thing.	Socialize the concerns and limitations and generate collective alternatives (2).
Ambivalent emotional effects	The non-presence in the classes and the access to other ways of teaching and learning, such as the inverted classroom, puts students in contradictory situations	Feel on contradiction
	If you care, you go to the class and listen to it, and you learn the same	The concentration depends if the student care not only of virtual conditions (2)
	The most positive aspects: “tutorials” and lectures who uploaded some videos and. that’s also a great thing because you can watch them whenever you want. There is less contact, the contact is worse, expressing yourself is scary.	Virtual resources facilitate learning autonomy and affect self-expression
	Not being able to live the academic experience in a university in Latin America was a bummer, but at the same time, I lived some things that I don’t think I will ever live again in my life.	The pandemic generates limitations on the inside and the outside, but new experiences beyond the university open new, unexpected possibilities (3).

Table 3 shows the emotional effects that the situation of confinement and isolation has generated in the students. In general, the adverse effects stand out, which are evident and have been named by the research: feeling nervous and overloaded, abandoning learning situations due to lack of attention, decreasing performance, feeling that isolation reduces interaction with others and affects relational experiences, feeling distracted, disconnected, and lacking concentration; seeing how it affects effort and retention to learn; feeling dispersion, virtual symbolic violence, embarrassment and anxiety. However, the decrease in relationships with others and the loss of agency stand out the most, producing, as Lisa points out, that one ends up being “a bit robotic”.

Nevertheless, if these are the predominant dimensions that require intense psychological support and pedagogies of care (Goralnik et al., 2012; Desierto and De Maio, 2020; Burke and Larmar, 2021; Mehrotra, 2021), the students have also been resilient and have generated alternatives to the situations they have had to live.

Some students feel more self-confident. It has opened their empathy with the teachers. They have learned to organize themselves, to generate alternatives to the limitations of the virtual classroom. They have generated actions of collaboration and care with their colleagues, and it has made self-knowledge possible.

However, the effects do not only move in a duality. Students have also experienced ambivalent situations associated with the contradictions in which they live, experiences of maladjustment to which they have had to adapt. They stated that the lack of concentration is not a determinant of the pandemic but a person’s decision. Most participants were aware of phenomena studied by different authors (Alter, 2017; Carr, 2020; Desmurget, 2020; among others) related to how digital devices capture and dispersing their attention and make it difficult to concentrate. Although virtuality also provides resources that facilitate learning and promote autonomy, above all, the pandemic generates limitations. However, they also point to new experiences beyond the university that unexpectedly open possibilities. Pau, an architecture student who spent part of the pandemic in Chile, synthesized this last consideration in the experience lived in the period when the democratic constituent process was opening, pointing out what may be a summary of students’ assessments of the emotional effects generated in their lives and the university:

Pandemic sucks; obviously, it always will. Online classes suck. Just like missing classes sucks, not being able to go to university sucks. Not living the academic experience at a university in Latin America was a bummer. Still, at the same time, I experienced some things that I don’t think I will ever experience again in my life (Pau).

The analysis on how the situation has affected students relationships with themselves, with others and with the world allows us to notice that after an initial moment in which virtual encounters seemed to be a continuation of their experiences before the pandemic, three responses emerged: blocking, adaptation, and reinvention. Researching the experiences of young university students during the pandemic time, based on in-depth interviews, makes it possible to encounter the unknown and feel challenged on how to make sense of it from the adopted onto-epistemic-methodological and ethical framework.

## Conclusion and Perspectives

When the COVID19 pandemic was confirmed and the lockdown occurred, different authors and organizations undertook urgent research to understand how the new situation affected university students’ lives. As reported in Khan’s (2021) meta-analysis, most of this research used questionnaires. In this same period, we initiated the TRAY-AP research project on trajectories and learning ecosystems of young university students. The meetings, despite the difficulties, were face-to-face. From the beginning, we introduced how they were dealing with the pandemic context and how the changes were affecting them in their daily lives and their relationship with the non-presential modes that university education offered them. Here, we started to see layers of young people’s experiences not considered in research based on questionnaires and the richness involved in implemented contextual and in-depth explorations. Learning is a deep contextual and process (Phillips, 2014) that occurs throughout, in the length, breadth and depth of life (Banks et al., 2007). The contextual and ongoing nature of learning reinforces the need to study educational phenomena in-depth and breadth, taking into account the experiences of those involved. Our initial challenge was to open a dialogue with the transcripts of the four conversations we have had with each student to appreciate how the inability to attend classes physically affected them emotionally in their relationship with their ways of learning at the university. The process we followed is explained in detail in the article and led us to identify seven key concepts around which the students’ accounts of their experiences converged. With one of these concepts, the one that had elicited the most significant number of scenes (# The emotional effect of virtual teaching and learning -35), we carried out a second-level analysis that allowed us to name the emotional effects the students had indicated. This new analysis allowed us to point out the most relevant contributions of the study.

The first consideration is the importance of carrying out research that followed the students in their movements and did not lead them to a previously defined goal by the researchers and only to answer given questions. The meetings held were for the students a place of calm, welcome and care. An opportunity to think about a subject (how and where with who and what they learn). Something “that is always there, but that I have not stopped to reflect on” (as Marc said). In this framework of relationships, the experiences around the lockdown opened up as an opportunity for listening (Les Back, 2007) and exploring different ways of narrating their experiences and allowing us to avoid getting involved in “extractive” research (Wilmsen, 2008) and engaging in an educational, inclusive and participatory (Elliott, 1988; Nind, 2014; Abma et al., 2019) process with participants. This scenario calls for educational research more embedded in people’s “real” lives and circumstances, considering their voices and their effects. The emerging knowledge might help teachers see students in a more complex and stuational way to improve their educational relationships and student learning.

As underlined in different reports and research on state of the art, the second consideration is that the university and the faculty, in general, were not prepared for such an abrupt change. Most of them emphasized the continuity of classes in virtual and distance format and not on the accompaniment and care of students in this transition. When students point out the emotional effects highlighted in the article, they point out how they had to face loneliness and caring relationships with other colleagues. The virtual has acted in urgency, but learning, as the students point out, is also an emotional experience and as Maria pointed out, “to learn the first thing I have to do is to take care of myself.” What raises this reflection is that universities should further consider how they can be institutions that take care of their members. We need more research based on the educational context and oriented toward improvement and change.

The third shows the difficulties highlighted by many participants to organize themselves and develop independent and self-regulated learning strategies. Students contributions reveal that higher education is mainly teacher-centered. Many universities still believe that “teaching is telling, learning is listening, and knowledge is what is in books” (Cuban, 1986, p. 27). In this way, moving face-to-face to virtual teaching usually becomes a set of lectures (sometimes broadcasted) followed, sometimes, by a set of questions. Most students lack skills and intellectual autonomy when other teaching methods (as flipped classroom or collaborative tasks). Contemporary higher education institutions operate in a highly complex and multifaceted world, where “data is cheap, but making sense of it is not” (Boyd, 2014). The great challenge in this context is how to provide young people with educational experiences that help them develop their full capacities to become responsible citizens of a better world. An ideal that is unattainable without the full participation and engagement of institutions, teaching staff and students.

The fourth consideration, as suggested above, relates to the need to pay attention to learners’ social and personal circumstances. Learning is a highly contextual process (Phillips, 2014), not just a cognitive process, but a fully embodied and experiential journey, in which affect, what affects us, plays a crucial role. The participants in our study with better living conditions and higher social and cultural capital found much fewer difficulties during the pandemic. They showed much more open perspectives and resources than others with different circumstances, as in the case of Blai, an Economics student, passionate reader, and highly engaged in cultural, social and political issues that remember the confinement time as very positive. “It was like a time of downtime that I could dedicate myself to reading, watching movies, and dedicating myself to what I liked without having any worries.” In this regard, the university cannot go on thinking of them as an equalizer. Not recognizing students’ diversity and not paying attention to their needs by treating all the “in an equal manner” can perpetuate the academic, social and cultural divide. This insight is of paramount importance in research about educational “excellence” and competitive rankings facing universities.

In conclusion, after the abrupt shock of the first confinement and the need to adapt without preparation or resources to a new university, work and social situation, students have, in general, developed emotional strategies and tactics of adaptation to this unexpected life condition. The consequences and lessons learned could better prepare them to better understand and cope with their present and future life in an ever-changing world. One of the remaining questions, which calls for further research, is if universities have learnt from this situation. Whether they are coming out of this crisis wiser and ready to make a difference, or whether they will be back to the “same” starting point.

## Data Availability Statement

The raw data supporting the conclusions of this article will be made available by the authors, without undue reservation, provided that they do not violate the ethical commitment.

## Ethics Statement

The studies involving human participants were reviewed and approved by Comissió de Bioética, University of Barcelona. The patients/participants provided their written informed consent to participate in this study.

## Author Contributions

Both authors contributed to the article and approved the submitted version.

## Conflict of Interest

The authors declare that the research was conducted in the absence of any commercial or financial relationships that could be construed as a potential conflict of interest.

## Publisher’s Note

All claims expressed in this article are solely those of the authors and do not necessarily represent those of their affiliated organizations, or those of the publisher, the editors and the reviewers. Any product that may be evaluated in this article, or claim that may be made by its manufacturer, is not guaranteed or endorsed by the publisher.

## Footnotes

1 Learning trajectories of young university students: conceptions, strategies, technologies, and contexts (https://cutt.ly/NRAFGZH).

2 Number of times participants refer to a given key concept.
